# Soil Bacterial Communities From the Chilean Andean Highlands: Taxonomic Composition and Culturability

**DOI:** 10.3389/fbioe.2019.00010

**Published:** 2019-02-05

**Authors:** Felipe Maza, Jonathan Maldonado, Javiera Vásquez-Dean, Dinka Mandakovic, Alexis Gaete, Verónica Cambiazo, Mauricio González

**Affiliations:** ^1^Laboratorio de Bioinformática y Expresión Génica, Instituto de Nutrición y Tecnología de los Alimentos, Universidad de Chile, Santiago, Chile; ^2^Center for Genome Regulation, Santiago, Chile

**Keywords:** atacama desert, altiplano highland, bacterial community, bacterial isolation, NGS, PGP

## Abstract

The Atacama Desert is a highly complex, extreme ecosystem which harbors microorganisms remarkable for their biotechnological potential. Here, a soil bacterial prospection was carried out in the high Altiplano region of the Atacama Desert (>3,800 m above sea level; m a.s.l.), where direct anthropogenic interference is minimal. We studied: (1) soil bacterial community composition using high-throughput sequencing of the 16S rRNA gene and (2) bacterial culturability, by using a soil extract medium (SEM) under a factorial design of three factors: temperature (15 and 30°C), nutrient content (high and low nutrient disposal) and oxygen availability (presence and absence). A total of 4,775 OTUs were identified and a total of 101 isolates were selected for 16S rRNA sequencing, 82 of them corresponded to unique or non-redundant sequences. To expand our view of the Altiplano landscape and to obtain a better representation of its microbiome, we complemented our Operational Taxonomic Units (OTUs) and isolate collection with data from other previous data from our group and obtained a merged set of OTUs and isolates that we used to perform our study. Taxonomic comparisons between culturable microbiota and metabarcoding data showed an overrepresentation of the phylum Firmicutes (44% of isolates vs. 2% of OTUs) and an underrepresentation of Proteobacteria (8% of isolates vs. 36% of OTUs). Within the Next Generation Sequencing (NGS) results, 33% of the OTUs were unknown up to genus, revealing an important proportion of putative new species in this environment. Biochemical characterization and analysis extracted from the literature indicated that an important number of our isolates had biotechnological potential. Also, by comparing our results with similar studies on other deserts, the Altiplano highland was most similar to a cold arid desert. In summary, our study contributes to expand the knowledge of soil bacterial communities in the Atacama Desert and complements the pipeline to isolate selective bacteria that could represent new potential biotechnological resources.

## Introduction

Soils are an extraordinarily diverse microbiome (Torsvik and Øvreås, 2002; Philippot et al., 2013). Microorganisms harbored in these environments have crucial roles in plant growth promotion and nutrient cycling (Fierer, 2017). Among, the Atacama Desert is often considered one of the harshest soil environments, displaying a spectrum of variables make it an extreme environment (Costello et al., 2009). It is the most arid desert on Earth (Gómez-Silva et al., 2008), it exhibits severe day-night temperature fluctuations (McKay et al., 2003; Azua-Bustos et al., 2012), and the highest UV radiations ever measured on Earth (Piacentini et al., 2003; Cordero et al., 2014), these environmental conditions are exacerbated in the highlands from the Altiplano region of the Atacama Desert.

The Andean Altiplano is a volcanic-sedimentary plateau located between 28 and 24°S with an average altitude of 4,000 m a.s.l. This environment is the second highest Altiplano region on Earth and is characterized by low mean annual temperature (<10°C) and precipitation (<170 mm), making it a cold arid environment within the Atacama Desert (Díaz et al., 2016).

Extremophile microorganisms that exist in environmental conditions like the Atacama arid highlands have unique metabolic capacities and/or physical structures which allow them to survive in these oligotrophic conditions (Tse and Ma, 2016). It is intriguing to understand how these microorganisms use various substrates and metabolic pathways to endure and how this undiscovered chemical diversity could be used for biotechnological applications (Bull and Asenjo, 2013).

Microbial communities in the soils of the Atacama Desert have been well-studied, because this region is considered the dry edge for life (Azua-Bustos et al., 2012; Azua-Bustos and González-Silva, 2014) and for its similarities with the soils of Mars (Navarro-González et al., 2003). The annual output of research publications on Atacama microbiology increased 10-fold during the last decade, reflecting an increasing interest in both fundamental and applied topics (Bull et al., 2016, 2018). However, most of the analyses have been performed with the use of molecular-genetic methods, and the study of isolates has mainly centered on members of the taxonomically distinct *Streptomyces* clade isolated from the Atacama Desert (Okoro et al., 2009; Rateb et al., 2011; Schulz et al., 2011; Goodfellow et al., 2017). Also, the studies mentioned have been mainly performed in soils from the lower parts of the Desert. Little is known about the diversity of bacterial communities associated with the Andean highlands (Lugo et al., 2008; Ferrero et al., 2010; Jorquera et al., 2016; Menoyo et al., 2017), which is precisely what we aim to uncover. We hypothesize that bacterial communities harbored in the highlands from the Atacama Desert display new candidates with biotechnological potential. To achieve our aim, we performed a soil bacteria prospection of three sites in the Atacama Desert highland and then tested the isolates for plant growth proportion properties (PGP) to evaluate the biotechnological potential harbored in this extreme environment.

The sampling sites of this study were between 3,800 and 4,500 m a.s.l., in the Altiplano highlands, nested below the Lascar Volcano. Lascar is the most active volcano of the northern Chilean Andes, with frequent small to medium eruptions and sporadic explosive events accompanied by ash rain extending over wide areas in Chile (Risacher et al., 1999; Tassi et al., 2009). Despite its extreme conditions, a diverse group of bacteria have been found to reside in the Lejía Lake, located at the base of Lascar Volcano (Demergasso et al., 2010; Mandakovic et al., 2018a). Thus, Altiplano highlands, especially close to the permanent influence of chemical components generated by volcano activity, constitute a relevant model to investigate how organisms adapt to live under particular extreme conditions.

As a first approach, we carried out a soil bacterial prospection using NGS technology to examine the bacterial community present in the Altiplano highland (>3,500 m a.s.l.) in the central region of the Atacama Desert. Once bacterial metabarcoding data was acquired, we evaluated bacterial culturability, using a soil extract medium (SEM) (Mandakovic et al., 2018a) under a factorial design of three factors: contrasting temperatures (15 and 30°C), nutrient contents (high and low nutrient disposal) and oxygen availability (presence and absence).

In the course of this study we uncovered part of the microbial biodiversity using these two different approaches. The combination of cultivation techniques coupled with high throughput sequencing allowed us to describe the microbial landscape of the high altitude areas of the Atacama Desert, and also to recover a part of the culturable fraction of the microorganisms inhabiting this natural extreme habitat.

Taxonomic comparisons between culturable microbiota and metabarcoding data showed overrepresentation and underrepresentation of different taxa that could grow in culture, revealing several candidates that may provide support for the biotechnological applications of soil bacteria species living in a stressful environment that is highly challenging for life.

## Materials and Methods

### Site Description, Sample Collection and Processing

The sampling sites were located in highlands of Altiplano region of the Atacama Desert (between 23°30′0.0″S 67°41′24.0″W and 23°19′42.8″S 67°47′56.0″W), located between 3,870 and 4,480 m a.s.l. Soil samples were obtained from two sampling sites: upper highland (4,480 m a.s.l.) and lower highland (3,870 m a.s.l.). Upper and lower highlands correspond to the TLT01 and TLT08 sites, respectively, of the Talabre-Lejía Transect (TLT; Díaz et al., 2016). Samples were taken in April, 2014, and again on April, 2017. Mineral soil samples (~200 g each) were randomly obtained in sterility between 5 and 10 cm deep in the soil and were used for the metabarcoding analysis, preparation of Soil Extract Medium (SEM), bacterial isolation and for physicochemical analyses. This depth was chosen as it has minimum effect of superficial phenomena like wind and rain with the highest microbial diversity (Eilers et al., 2012). Samples were kept at 4°C for isolate culture and −20°C for metabarcoding, and transported to the laboratory, where they were kept at the same temperature and processed a few days after sampling.

### Local Environmental Measurements and Soil Physicochemical Analyses

Soil physicochemical characteristics, pH and electrical conductivity were retrieved from Mandakovic et al. (2018a,b). Furthermore, environmental mean annual temperature (MAT) and mean annual precipitation (MAP) of each site were taken from Díaz et al. (2016). Solar radiation was measured using the *in situ* insolation sensor S-LIB-M003 (Table 1).

**Table 1:** Average climatic and soil characteristics of the sites included in this study.

**Sampling location**	**Latitude °S**	**Longitude °W**	**Elevation (m)**	**pH**	**MAP**	**MAT**	**Electric conductivity (mS/cm)**	**%C**	**%N**
LLS^*^	−23.83	−68.15	4,314	8.5	161.9^**^	8.5	ND	2.43 × 10^−3^	3.98 × 10^−4^
TLT1^†^	−23.50305	−67.72371	4,480	5.23	161.9	4.2	0.06	0.16	0.01
TLT8^†^	−23.32856	−67.79890	3,870	5.77	75.1	6.9	0.04	0.32	0.02

* Lejía Lake Soil (Mandakovic et al., 2018a);

** MAP from LLS was extrapolated from TLT1 given the proximity of the sites;

† *TLT, Talabre-Lejía Transect (Díaz et al., 2016); ND, No Data*.

### Soil DNA Extractions and Sequencing

Soil DNA was extracted according to Mandakovic et al. (2018a) with some modifications. Total DNA from soils was extracted from 10 g of each sample using the NucleoSpin® Food kit (Macherey-Nagel), following the manufacturer's instructions, and the CTAB extraction buffer published by Zhou et al. (1996). Microbial 16S rRNA genes were amplified according to Mandakovic et al. (2018b) without modifications. DNA libraries were constructed following the TruSeq DNA sample preparation (Illumina, USA) protocol. Sequencing was performed by MrDNA Next Generation Sequencing Service Provider (Shallowater, Texas, USA) on an Illumina MiSeq platform in an overlapping 2 × 300 bp configuration, to obtain a minimum throughput of 40,000 sequences (reads) per sample.

### Metabarcoding and Isolate Complementary Data

In order to obtain a broader representation of the highland microbial community, the data obtained in this study was complemented with metabarcoding data and isolate collection from Mandakovic et al. (2018a,b). Metabarcoding data from TLT8 and Lejía Lake shore were taken from Mandakovic et al. (2018a,b) respectively, and merged with the data of TLT1 obtained in this study. To represent the Lejía Lake shore soil among the isolates collection, we included the bacterial isolates recovered by Mandakovic et al. (2018a) in this study as well.

### Processing of Illumina Sequence Data

Microbial 16S rRNA raw amplicon sequences were processed and analyzed following previously described protocols (Dowd et al., 2008; Handl et al., 2011). Briefly, sequences were joined (overlapping pairs) and depleted of barcodes. Then, sequences <150 bp or with ambiguous base calls were removed. Sequences were filtered using the USEARCH clustering algorithm at 4% sequence divergence to remove chimeras and clusters consisting of only one sequence (i.e., singletons) (Edgar, 2010; Edgar et al., 2011). Finally, sequences were quality filtered with Mothur v.1.22.2 (Schloss et al., 2009) with the minimal quality average set to 30.

### Accession Numbers

All 16S rRNA gene sequence data used in this study are deposited in the Sequence Read Archive (SRA) of the National Center for Biotechnology Information (NCBI) under the BioProject accession number PRJNA489888.

### Sequence Analysis and Taxonomic Identification Using the Silva Database

Following Mandakovic et al. (2018b), sequences were analyzed with the software Quantitative Insights Into Microbial Ecology (QIIME v1.8.0; Caporaso et al., 2010). We used QIIME script “pick_closed_reference_otus.py” to extract all 16S rRNA reads from the amplicon data that matched Silva r16S database (Quast et al., 2012) (release 132) at 97% similarity or 3% divergence, with the taxonomy of the resulting Operational Taxonomic Units (OTUs) assigned directly from the closest reference sequence match. The OTU picking process was done with USearch v6.1.544 (Edgar, 2010; Edgar et al., 2011) using QIIME default parameter values (-s 0.97 –z True –max_accepts 1 –max_rejects 8 –word_length 8 –minlen 64 –usearch61_sort_method abundance). OTUs unassigned or assigned to mitochondria and chloroplasts were removed.

### Alpha Diversity Analysis

We calculated alpha OTU diversity by randomly sub-sampling (without replacement) each soil sample using the alpha_rarefaction.py script in QIIME. The Chao1, Shannon, Faith's phylogenetic diversity (PD) and Evenness indices were calculated, along with the observed number of OTUs. Rarefaction curves for each of these metrics were obtained by serial subsampling in increments of 4,199 sequences and 10 iterations per increment, to a standardized 42,000 sequences per sample.

### Factorial Culture Design

For culture experiments, Soil Extract Medium (SEM) was used and prepared according to Mandakovic et al. (2018a). Briefly, SEM was prepared by mixing agar with the water soluble portion of the soil from the sampling sites, supplemented with Fungizone (2.5 μg/ml). This medium was either: (1) supplemented with Lysogeny Broth (LB-SEM; 10 g/l Bacto Tryptone (BD), 5 g/l Yeast Extract (BD), 10 g/l NaCl); or (2) supplemented with Diluted Lysogeny Broth (10% LB-SEM; 1 g/l Bacto Tryptone (BD), 0.5 g/l Yeast Extract (BD), 1 g/l NaCl). The pH of these media was adjusted to pH 7. Each medium was incubated in a combination of 2 conditions: temperature (15 or 30°C) and oxygen availability (aerobic or anaerobic conditions). This factorial design of three factors returned the eight conditions used in this study, and offered a wider range of conditions that the highland environment displayed, to isolate a higher diversity of soil bacteria.

### Isolation and Identification of Unique Bacteria

Collected samples were passed through a sterile 2 mm sieve for homogenization. To obtain soil bacterial isolates from the Altiplano region of the Atacama Desert, 1.5 g of sieved-homogenized soil was solubilized in 2 mL sterile PBS for 2 h. Tubes were then centrifuged for 5 min at 500 rpm and 100 μl of supernatant were plated in triplicate and cultured for 2 weeks in the combination of conditions previously described. A total of 101 morphologically different colonies were isolated. Bacterial DNA from these isolates was extracted and purified using the DNeasy Blood & Tissue Kit for DNA (Qiagen). The 16S rRNA genes were amplified by PCR using the primers 27F (5′-AGA GTT TGA TCA TGG CTC AG-3′) and 1492R (5′-CGG TTA CCT TGT TAC GAC TT-3′) according to Mandakovic et al. (2018a). PCR products were visualized in 2% (w/v) agarose gel electrophoresis in Tris-acetate-EDTA (TAE) buffer (1X) and stained with ethidium bromide. PCR products were purified and sequenced by Macrogen (Macrogen Corp., Maryland, USA). Forward and reverse sequences were assembled in a single contig using the Contig Assembly Program in the Bioedit software. The phylogenetic classification of these sequences was determined using the RDP database tools (Cole et al., 2014). Sequence identity was then determined comparing the 16S sequence of all the isolates obtained using the TaxonDC software (Tarlachkov and Starodumova, 2017). Those exhibiting higher than 99% sequence similarity and also identical morphological features were collapsed to one.

### 16S rRNA Phylogenetic Analysis

For phylogenetic analyses of the unique 16S rRNA sequences recovered, reference species sequences and outgroups were obtained from the Silva database. Sequences were aligned using the MAFFT v7.313 software using the default (FFT-NS-2) algorithm and subsequently trimmed and realigned using the G-INS-I algorithm. Phylogenetic trees were constructed using MEGA7 software with the following settings: Maximum likelihood tree, Tamura-Nei model, with 1,000 bootstrap replications. The trees were visualized and annotated using the FigTree Software^1^.

### Determination of Biotechnological Applications

Bacterial isolates were taxonomically matched to known species using the RDP database (Supplementary Table 1). They were studied in depth in order to describe their biotechnological potentials by literature search using the NCBI Pubmed database. The main keywords used were the “genus” and “species” assigned for each isolate, and the keyword “review” was added when more than 200 results were retrieved with the aforementioned keywords (e.g., “*Streptomyces vinaceus*,” “*Bacillus megaterium* review”). Articles were taken into account if they declared any kind of biotechnological potential information in their abstract. Functions described in published articles were classified as: “Biomedical potential,” “Bioremediation,” “PGP,” “Agricultural potential,” “Food industry potential,” “Pharmaceutical Industry potential,” among others.

### Identification of Plant Growth-Promoting (PGP)

To classify the isolates as plant growth-promoting (PGP), we evaluated 5 attributes (siderophore production, Indole Acetic Acid (IAA) production, nitrogen fixation, 1-Aminocyclopropane-1-Carboxylate (ACC) deaminase activity and phosphate solubilization), each with different selective culture media. Siderophore production was detected using the method described by Schwyn and Neilands (1987). Using chromeazurol agar (CAS), bacterial colonies that exhibited a yellow halo after proliferation were classified as positive for siderophore production. IAA production was assessed using a colorimetric method. Bacterial isolates were cultured in TSB media supplemented with 0.1% L-tryptophan as the precursor for IAA. Using Salkowski's reagent (Mohite, 2013), TSB media acquires color depending on the IAA concentration, which is then compared to a standard curve of Indole-3-acetic acid (Merck®) ranging from 5 to 180 μg/ml. Nitrogen fixation was determined using Nitrogen-Free Medium (NFM). Isolates were incubated for 7 days, and bacterial proliferation was evidence of atmospheric nitrogen fixation (Grobelak et al., 2015). ACC deaminase activity was measured by culturing bacteria with 3 mM of 1-Aminocyclopropane-1-carboxylic Acid (ACC; Merck®) as the sole source of nitrogen (Penrose and Glick, 2003). The phosphate-solubilizing ability was determined by the presence of a halo surrounding colonies in Pikovskayas Agar medium after 4 days of cultivation at 30°C (Ahmad et al., 2008).

## Results

### Sampling Location and Soil Characterization

The highland sampling sites used in this study were Altiplano soils defined as the upper highland (TLT1: 4,480 and LLS: 4,314 m a.s.l.) and the lower highland (TLT8: 3,870 m a.s.l.). Physicochemical features of each site are displayed in Table 1. Average physicochemical features of the sampled soils were: electric conductivity (mS/cm) 0.8 ± 0.6, pH 6.2 ± 1.3, MAT was 5.5°C, MAP 118.5 mm, 0.16% C and 0.01% N. The solar radiation reported by the insolation sensor station at 4,090 m a.s.l. was 960 W/m^2^. According to the MAP value, highlands from the Atacama Desert are classified as an arid environment, less arid than the Atacama hyper-arid core at 2,500 m a.s.l. Considering the high altitude of the Lascar Volcano, this area is seasonally covered by snow, which contributes to decrease the MAT and increase MAP. Also, according to Díaz et al. (2016) the highest plant coverage (%) is reached at 4,000 m a.s.l. a feature that is probably driven by the higher MAP of this highland environment.

### Bacterial Community Composition and Diversity Analysis

To identify the complete bacterial community (microbiome) present in the soil, we used NGS technologies to sequence the 16S rRNA gene directly from the environment. A total of 417,671 good quality (clean) reads were obtained, of which 151,245 were classified in a 16S rRNA sequence (mapped reads). The total number of different OTUs recovered from soil microbiome were 9,961, with only 1,211 (12,2%) containing >80% of the relative abundance, showing a typical power law distribution pattern found in most soil microbial communities (Bailey et al., 2013). After applying a quality filter of 0.005% of the total read abundance, the number of OTUs was reduced to 4,775. The minimum taxonomic level assigned to each OTU was phylum; 53.3% were assigned to genus and 6.6% to species.

Soil bacterial community alpha diversity was calculated as indicated in material and methods. We obtained Shannon diversity values of 9.5 ± 0.0 in the upper highland, 10 ± 0.0 in the lower highland and 6.7 ± 0.0 for Lejía Lake shore. The estimated richness values (Chao1) were 7,324.7 ± 88.3 for the upper highland, 8,351.5 ± 149.5 for the lower highland and 3,212.4 ± 43.5 for Lejía Lake shore. The mean diversity (H') of the highland was 8.8 ± 0.0 and the mean richness (Chao1) was 6,296.2 ± 93.8. Soil bacterial diversity appeared to be responding positively to altitude, since we obtained higher diversity values in upper highland sampling sites. It appears that lake soil limits the diversity because it is located at the same altitude than the upper highland but harbors less diversity levels.

A total of 37 bacterial phyla were found in these soil samples, Proteobacteria (36.52%) being the most abundant (dominant), followed by Acidobacteria (20.82%), Actinobacteria (17.42%), and Bacteroidetes (5.93%). Of the 4,775 OTUs, 2,547 were identified to genus, representing 480 different genera (Supplementary Figure 1).

### Characterization of Culturable Bacteria

The community characterization allowed us to have a vision of the bacterial community inhabiting highlands from the Altiplano region of the Atacama Desert. Nevertheless, since bacterial physiological characterization is primarily achieved in culture, we applied culture-based approaches that simulated the natural conditions and permitted the identification of the complete set of bacteria recovered in culture. To accomplish this, we used several culture conditions, described in methods. These culture conditions were proposed to cover a wide range of bacterial growth conditions and isolate a high variety of microorganisms, and the use of SEM intended to generate a medium that could represent many qualities encountered in Altiplano soils by using the nutrients present in the water-soluble fraction obtained directly from the soil.

About 1,000 bacterial isolates were recovered from the initial culture, from which 101 were selected as morphologically unique. The 16S rRNA of these isolates was sequenced and their sequence similarity was evaluated using the TaxonDC software in order to collapse identical sequences. From the 101 isolates, a total of 82 were then selected as unique phylotypes by their 16S rRNA gene sequence and morphological features. Isolates recovered by Mandakovic et al. (2018a) (*n* = 11) were added to this pool of 82 isolates. Further analyses of phyla abundance were determined with the 93 bacterial isolates recovered in the Altiplano highland (Supplementary Table 1).

**Figure 1:**
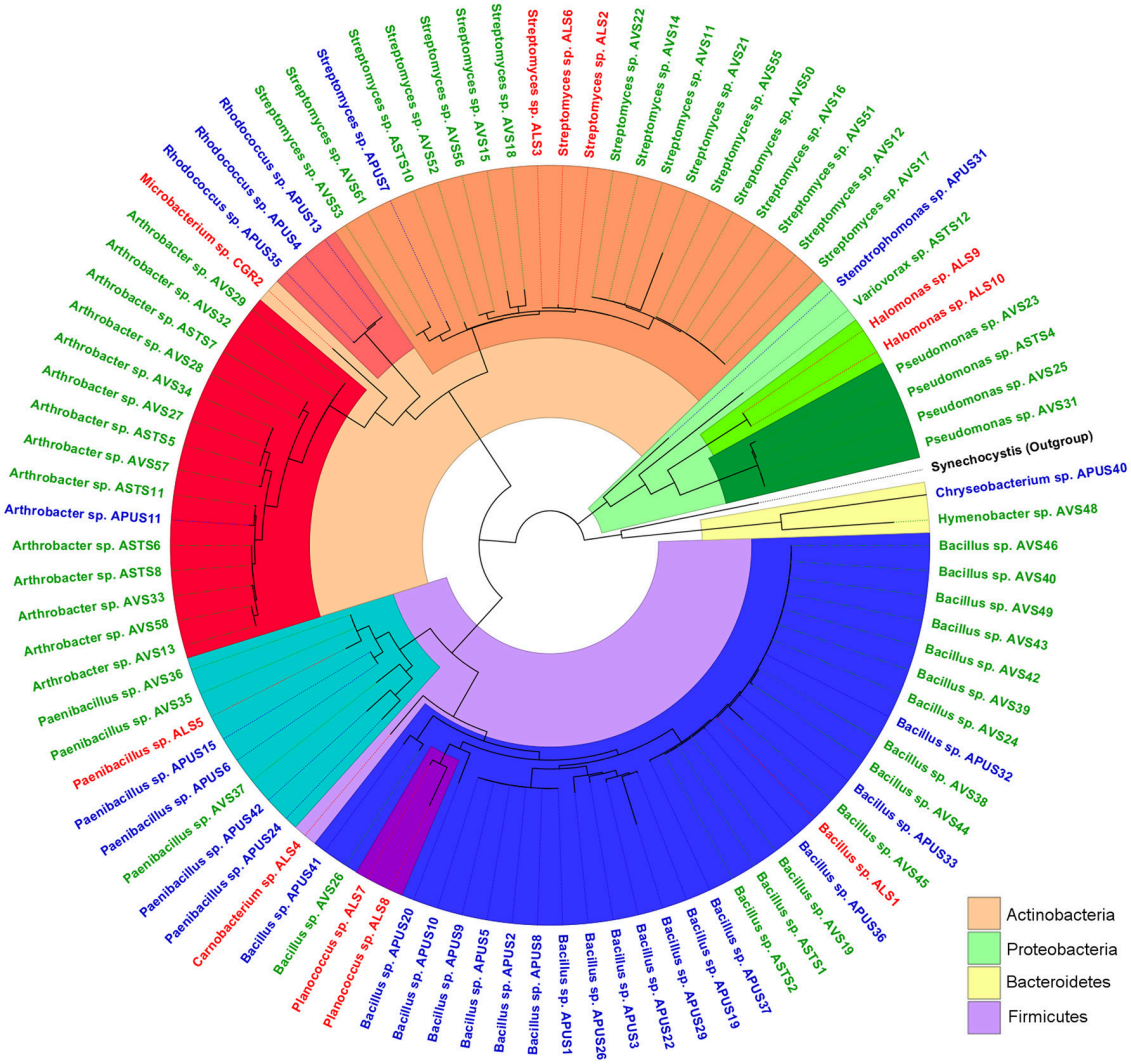
Phylogenetic tree of the bacterial isolates recovered from the highlands of the Atacama Desert. Colors from the inner circle represent the phyla and genera of the bacterial isolates: Proteobacteria (green color range), Firmicutes (blue color range), Actinobacteria (orange color range), and Bacteroidetes (yellow); label color represents the isolation site: Lejía Lake (red), TLT1 (green), and TLT8 (blue). Synechocystis was used as outgroup and marked in black. The tree was constructed using the MEGA7 software and edited with the FigTree software.

Culturing revealed four phyla present in the soil bacterial community (Figure 1). The most abundant was Firmicutes (46.2%), followed by Actinobacteria (43%), Proteobacteria (8.6%), and Bacteroidetes (2.2%). These four phyla comprised 14 genera: the most abundant were *Streptomyces, Bacillus*, and *Arthrobacter* (Table 2). Interestingly, we isolated one member of the genus *Variovorax*, which has only seven other species described in its group.

**Table 2:** Isolates categorized by their genus assigned by 16S rRNA similarity index and isolated location.

**Genus**	LLS^*^	**TLT1^†^**	**TLT8^†^**	**Total**
Arthrobacter		14	1	15
Bacillus	1	14	17	32
Chryseobacterium			1	1
Hymenobacter		1		1
Paenibacillus	1	3	4	8
Pseudomonas		4		4
Rhodococcus			3	3
Stenotrophomonas			1	1
Streptomyces	3	17	1	21
Variovorax		1		1
Microbacterium	1			1
Carnobacterium	1			1
Planococcus	2			2
Halomonas	2			2

* Lejía Lake Soil (Mandakovic et al., 2018a);

† *TLT, Talabre-Lejía Transect*.

Contrary to what could be expected, not all 14 genera recovered by culture were identified by NGS. Though, only 4 genus were not recovered: *Carnobacterium, Chryseobacterium, Hymenobacter*, and *Rhodococcus*, they only represented 6 isolates. The culturable strategy recovered the same most dominant phyla found in the metabarcoding analysis (Proteobacteria, Actinobacteria, and Firmicutes). However, these phyla were represented in different proportions in the NGS results: the proportion of Actinobacteria was 2.5 times higher in culture and Firmicutes was 20 times higher than NGS. On the contrary, the culture dependent identification recovered four times less Proteobacteria than NGS. Moreover, no isolation of any Acidobacteria was accomplished.

### Putative Biotechnological Potential

**Figure 2:**
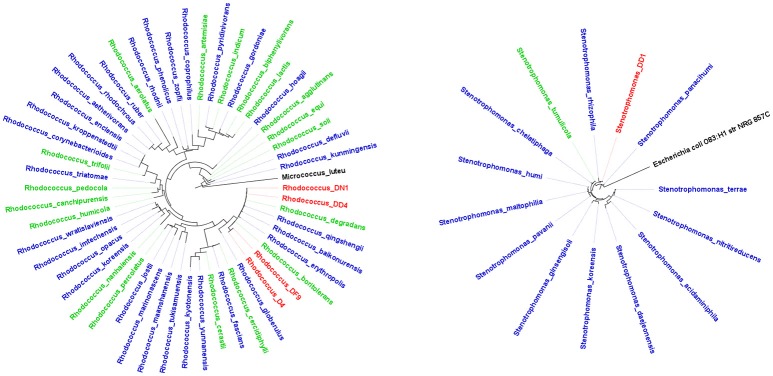
Maximum-likelihood tree of some of the bacterial genera that exhibited the highest number of biotechnological features. **(A)** *Rhodococcus* and **(B)** *Stenotrophomonas*. Blue and green labels represent complete (available at NCBI) and incomplete (partial sequences available at NCBI) genomes, red labels represent isolates recovered in this study and black labels represent the outgroups.

Bacterial isolates taxonomically identified to putative species using the RDP database (Supplementary Table 1) were studied in depth to describe their biotechnological potential using the NCBI Pubmed database. The most abundant categories of biotechnological potential found were Biomedical, Bioremediation and PGP (Table 3). Most genera evaluated had at least one species linked to the three most abundant categories, e.g., *Bacillus, Microbacterium, Paenibacillus, Rhodococcus, Stenotrophomonas*, and *Streptomyces*. However, *Arthrobacter, Halomonas, Pseudomonas*, and *Variovorax*, have only been described as potential PGP or to be involved in bioremediation. The genus *Bacillus* has been linked mostly to PGP, while the genus *Streptomyces* has generally been described as well-known antibiotic producers. Proportionately, *Rhodococcus* and *Stenotrophomonas* were the genera with more biotechnological features described in journal articles (Figure 2). Therefore, isolates from these genera are more likely to have the most biotechnological features.

**Table 3:** Most prevalent biotechnological capabilities among the 93 isolates according to the literature.

**Genus**	**Biomedical**	**Plant growth promotion**	**Bioremediation**
Arthrobacter		2	
Bacillus	3	8	6
Halomonas			1
Microbacterium	1	1	1
Paenibacillus	2	1	1
Pseudomonas		1	1
Rhodococcus	2	1	1
Stenotrophomonas	1	1	1
Streptomyces	9	1	1
Variovorax			1

The isolates obtained in this study were identified in terms of their 16S rRNA sequence and a putative species affiliation was assigned to each isolate based on the RDP database's Sequence Match algorithm. For the 93 isolates, 43 potential species were assigned. The isolation source of the representative strain for each isolated species was determined using the Global Catalog of Microorganisms (gcm.wfcc.info). Out of the 43 potential species, 40 were obtained from soil, rhizosphere or plants. The other 3 species were isolated from scleromata (*Arthrobacter scleromae*), airborne (*Hymenobacter aerophilus*), and food (*Carnobacterium* sp.), indicating a potential new source of species isolation. However, the assigned species were not corroborated by DNA-DNA hybridization, GC content or fatty acid profile, meaning that we cannot reach a definitive species identity.

Nevertheless, most of the bacterial references were isolated from soil and had biotechnological potential capabilities reported in NCBI. An *in silico* exploration was carried out in NCBI, and the most common potential capabilities recovered were: Biomedical, Bioremediation, and Plant Growth Promotion (Table 2).

### Biochemical Characterization of Bacterial Isolates

We screened 12 of our isolates for PGP activities (Supplementary Table 2); 12 of them were positive to at least 1 of the conditions tested, making them potentially PGP bacteria. Among these 12 isolates, none of them showed all the essayed PGP activities, nine correspond to siderophore producers (*Planococcus* sp. ALS8, *Planococcus* sp. ALS7, *Streptomyces* sp. ALS6, *Microbacterium* sp. CGR2, *Bacillus* sp. ALS1, *Pseudomonas* sp. ASTS4, *Stenotrophomonas* sp. APUS31, *Paenibacillus* sp. APUS6, *Bacillus* sp. AVS49), two solubilized phosphate (*Microbacterium* sp. CGR2, *Bacillus* sp. ALS1), six were potential nitrogen fixers (*Bacillus* sp. APUS41, *Pseudomonas* sp. ASTS4, *Stenotrophomonas* sp. APUS31, *Paenibacillus* sp. APUS6, *Bacillus* sp. AVS49, *Rhodococcus* sp. APUS4), four possessed ACC deaminase activity (*Streptomyces* sp. ALS6, *Bacillus* sp. APUS41, *Pseudomonas* sp. ASTS4, *Bacillus* sp. AVS49) and five were auxin producers (*Bacillus* sp. APUS41, *Pseudomonas* sp. ASTS4, *Paenibacillus* sp. APUS6, *Arthrobacter* sp. AVS27, *Bacillus* sp. AVS49). The strongest PGP potential was predicted in two isolates belonging to the genus *Pseudomonas* and *Bacillus*. PGP distribution across the different isolates revealed that the *Bacillus* sp. AVS49 and *Pseudomonas* sp. ASTS4 had the highest number of PGP potential activities, with four PGP potential traits, followed by and *Paenibacillus* sp. APUS6 with three different PGP potential traits.

## Discussion

Current interest in terraforming Mars has led to a series of studies of the “Mars-like” Atacama Desert (Bull et al., 2018). Formerly presented as the “dry limit of microbial life” (Navarro-González et al., 2003), the desert soil was considered devoid of living organisms. Today, however, bacterial organisms from the Atacama Desert have gained interest from the scientific community for their ability to survive and thrive in this extreme environment, and the biotechnological potential they harbor (Connon et al., 2007; Escudero et al., 2007; Lester et al., 2007; Cabrol et al., 2009; Demergasso et al., 2010; Neilson et al., 2012; Crits-Christoph et al., 2013; Idris et al., 2017).

The environment studied displayed unique features within the Atacama Desert biotope. It is arid, but not hyper-arid, because of the high average MAP of all the sites studied, making it more diverse for microbial communities than other sites, like the hyper-arid core of the Atacama Desert (Neilson et al., 2017). Because of the higher MAP values it also displays the highest plant coverage in the altitude gradient of the Atacama Desert landscape (Díaz et al., 2016). Given the altitude, it displays lower MAT than the hyper-arid core, located at 2,500 m a.s.l (Crits-Christoph et al., 2013). These features make the studied landscape a cold arid highland with different environmental and biotic conditions than the rest of the Atacama Desert and, therefore, possibly harbor a different bacterial community.

Our first approach to describe the microbial communities of the Altiplano highlands was through NGS. From 7,829 OTUs obtained, 34.4% were assigned to a genus, but only 4.4% to species. Thus, 30% of the OTUs, identified to genus, did not match any species in the GreenGenes database. The fraction of OTUs identified to genus in this study was high compared to others. The low proportion of genera that could be identified to species could be proof of the novel genetic potential present in the Atacama Desert. Considering the large area of arid biotopes and its insufficient study, it is likely that a large part of unexplored microorganism biodiversity is still hidden in these soils.

There was discordance between the bacterial phyla found by NGS and culture. Thirty seven bacterial phyla were found by NGS, while the isolates belonged only to four phyla. Moreover, the phyla isolated did not reflect the most representative phyla found in NGS. The difference between phyla diversity of NGS and isolates has been previously reported, particularly in hot deserts. Chanal et al. (2006) studying the Tataouine Desert by 16S rRNA sequence, found an overrepresentation of Bacteroidetes and Firmicutes and an underrepresentation of Firmicutes and Actinobacteria compared to the isolates they found. Abdul-Majid et al. (2016), studying Sand Dunes from the Qatari Desert, reported the absence of Chloroflexi among isolates, while in NGS this phylum had 2% relative abundance. They suggested that special conditions would have been needed in order to isolate this metabolic specialist group. They also found an overrepresentation of Bacteroidetes and Proteobacteria with NGS, in contraposition to Actinobacteria, which were underrepresented in molecular identification as well as in our NGS. Dunbar et al. (1999), studying the isolates of the Southwestern Desert in the USA, did not find Acidobacteria among their isolates, while this phylum was the most predominant in their massive sequence analysis. This evidence indicates that there is a bias when isolating bacteria from arid soils. This may be due to the differential supply of nutritional requirements in the culture media and in the soil, the removal of growth inhibitory components and/or the reduction of competitive interactions with members of the community, like competitive bacteria-bacteria interactions (Auld et al., 2013).

Different culture conditions also affected the bacterial diversity recovered. For example, in this study the *Arthrobacter* genus was mostly recovered under cold culture conditions, *Streptomyces* in hot culture conditions and the phylum Firmicutes was mostly recovered in media with low nutrient content. Other studies have focused on recovering rare taxa, e.g., optimizing media for isolating Acidobacteria, Actinobacteria, Proteobacteria, Verrucomicrobia, and the “Unculturable bacteria” (Janssen et al., 2002; Vartoukian et al., 2010; Campanharo et al., 2016). This information supports, in part, the discrepancy found between isolates and NGS.

As mentioned above, the Atacama Desert has been previously studied by several authors, but most studies have focused in the Yungay area, the hyper-arid core of the Atacama Desert (Connon et al., 2007; Lester et al., 2007; Navarro-Gonzalez et al., 2009; Neilson et al., 2012, 2017). Nevertheless, the Atacama Desert ranges from the Pacific Ocean to the highest areas of the Andes Mountain range, has several active volcanoes, arid to hyper-arid zones, etc.

The variety of these environmental conditions creates multiple niches for microorganism community development. Demergasso et al. (2010) studied physicochemical parameters and the microbial composition of water and sediment samples obtained from the saline Lejía Lake of the Atacama Desert. Remarkably, using NGS we were able to distinguish, in our merged set of OTUs all 6 bacterial phyla and 11 genera described by Demergasso and coworkers, together with 31 other mostly in low abundance. Similarly, Costello et al. (2009), studied the bacterial soil composition at Volcán Socompa (arid soil, 5,235 m a.s.l., pH 5.4), located 120 km to the South of Volcan Lascar and sharing similar environmental conditions. The predominant bacteria found belong to phyla Actinobacteria, Acidobacteria, Bacteroidetes, and Verrucomicrobia, with low abundance of Proteobacteria and Firmicutes. Except for the Proteobacteria, the other phyla show a similar pattern to the one found in our study. These bacterial community compositions reflect a pattern of microbial communities among Altiplano highland soils.

In the Yungay region, bacterial communities mostly exhibit a predominant abundance of Actinobacteria (between 70 and 94%), followed by Proteobacteria, Chloroflexi, Acidobacteria, and Firmicutes (Connon et al., 2007; Neilson et al., 2012; Crits-Christoph et al., 2013; Azua-Bustos et al., 2015). According to Maestre et al. (2015), Acidobacteria are inversely correlated with aridity, which explains higher abundance in our NGS OTUs than those reported in the hyper-arid core of the desert. Likewise, Neilson et al. (2017) state that Actinobacteria are mostly abundant in hyper-arid soils, which could explain the lower abundance of Actinobacteria found in the arid highlands. The bacterial community composition recovered in this study follows the trend portrayed in other studies of the Atacama Desert.

Fierer et al. (2012) stated that the microbial community profiles from desert environments were distinct from one another, but the differences observed between deserts were less than between desert and non-desert environments. Consequently, in order to compare the highlands of the Atacama Desert, we contrasted the diversity indexes (H' and Chao1) with other reports from arid environments. The most similar diversity index comes from Neilson et al. (2017), specifically in the highland sampling sites of the Atacama Desert. This result corroborates our methodology since the sampled environment was the same. Contrary to what could be expected, the second most similar diversity value, reported by McCann et al. (2016), comes from an Artic Desert in the Kongsfjorden region of Svalbard, Norway. Other articles about the Atacama Desert, including highlands (Demergasso et al., 2010) and hyper-arid areas (Neilson et al., 2012; Crits-Christoph et al., 2013), were included in the comparison as well, but returned lower diversity indexes than the aforementioned. This unexpected result can be attributed to the comparable environmental physicochemical conditions described by McCann et al. (2016) and those described in this study, i.e., low MAT, near neutral pH, MAP around 100–200 mm per year, and presence of seasonal snowfall (Supplementary Table 3). These conditions make the highlands of the Atacama Desert more closely related to Arctic Deserts than other hot and cold deserts, corroborating its cold arid environmental conditions.

The high diversity values presented in this study compared to others from the Atacama Desert, could be explained by the methodology adopted by each group. More replicates and high-throughput strategies would increase the diversity indexes, making this study more similar to Neilson et al. (2017), than Demergasso et al. (2010). However, aridity has been described as inversely correlated with bacterial diversity (Maestre et al., 2015; Neilson et al., 2017). Considering the highlands of the Altiplano as an arid environment, it would be expected to be more closely related to other distant arid environments than to neighboring hyper-arid ones.

Comparing the bacterial community composition of the Atacama Desert to hot deserts, including southwestern USA deserts and Volcán Socompa (Costello et al., 2009; Fierer et al., 2012), and cold deserts, including Antarctic dry valleys and Polar deserts (Supplementary Tables 4, 5; Pointing et al., 2010; McCann et al., 2016), we observed higher similarities to the bacterial community recovered by McCann et al. (2016) in Kongsfjorden (Arctic circle). This observation can be attributed to the environmental similarities previously mentioned. These environmental drivers seem more relevant to microbial community composition than altitude or geographic location. The statement presented by Lourens Baas-Becking in 1934, “Everything is everywhere, but the environment selects” (O'Malley, 2008) might apply in this scenario, only in terms of phylum composition, but the genera and species are probably unique to each environment.

Some authors have reported different biotechnological capabilities found among isolates in the Atacama Desert. Within the phylum Actinobacteria, in the genus *Streptomyces*, some species are currently used in the pharmaceutical industry for their antibiotic, antitumoral and neurodegenerative protecting molecules (Okoro et al., 2009; Nachtigall et al., 2011; Rateb et al., 2011; Leirós et al., 2015; Idris et al., 2017). On the other hand, the genus *Acidithiobacillus*, has potential in the biomining industry, while the genera *Bacillus* and *Micrococcus* are good antibiotic producers (Azua-Bustos and González-Silva, 2014).

The biotechnological traits found *in silico* highlights the effect that these isolates could have in the agricultural industry, considering the amount of species that were associated with PGP capabilities and the number of isolates that were positive to the PGP experiments. However, given the dimension and different environmental conditions found along the Atacama Desert, these capabilities may arise mostly in the arid areas, considering the absence of plants in the hyper-arid sectors of this desert.

Studies about PGP have been developed in different environments, including other cold deserts. In the cold Leh Ladakh deserts (Yadav et al., 2015), most of the psychrotrophic bacterial isolates evaluated, exhibited positive results to PGP, including members of the Firmicutes, Proteobacteria, Actinobacteria, and Bacteroidetes phyla. Another study of PGP competences amongst isolates was carried out in the Sahara Desert (Cherif et al., 2015). Most of the isolates exhibited some degree of PGP capabilities, but the most relevant phylum was Firmicutes, specifically the *Bacillus* genus, a genus that has been reported to exhibit consistent and diverse PGP capacities (Cherif-Silini et al., 2016). The associations between culturable microorganisms from extreme environments and plant growth promoting potential supports our findings, in which all the isolates tested displayed PGP potential. This absolute proportion is higher than the ones found in other desert soil studies and is supported by the fact that there is higher plant coverage (%) around our sampling altitude (4,000 m a.s.l.; Díaz et al., 2016) than at other altitudes in the Atacama, accepting the hypothesis that the foundations for the adaptability to the harsh conditions of plant growth in arid lands are based on the co-evolution of the association between plant and microbes under harsh environmental conditions. The PGP potential of the strains used in this study requires further evaluation, but this precedent supports our pursuit to find bacterial isolates with new biotechnological attributes in this extreme environment.

The difference in PGP potential traits representation between siderophore release and auxin producers has been documented before by Marasco et al. (2012). They suggest that soil bacteria have higher nutrient supply capacities than endophytes which exhibit higher auxin production, suggesting an environmental-driven differentiation in PGP activities. This same report by Marasco et al. (2012) also reports higher PGP activities in non-cultivated soils than cultivated ones. Additionally, a study from Israel showed a significantly higher population of potentially PGP bacteria in the stressful sunny site than in the shadowed site (Timmusk et al., 2011), supporting our search in this arid—uncultivated environment.

For the first time in environmental microbiology research, we linked the Altiplano highlands from the Atacama Desert with cold arid soils from the Arctic desert. Based on their similarities in microbial community composition and physicochemical and environmental features, we added evidence to the hypothesis that environmental features determine the microbial community composition, rather than geography or altitude.

We sampled a substantial proportion of the strains (43 unique phylotypes according to the RDP database) in a highland soil microbial community and were able to detect a variety of taxonomic groups typical from arid soils, assessing their phylogenetic distribution and their culturability.

NGS had better depth and detection limit analyzing bacterial diversity, revealing the rare bacteria present in the community without the bias of disclosing only the most abundant microorganisms. We demonstrated that there is novel genetic potential in the soils sampled. Over 30% of the bacterial sequences of the total number of OTUs found in the community were phylogenetically unique. These novel sequences may represent a previously unknown bacterial diversity that is endemic to this highly insular and harsh elevation ecosystem. This reinforces the need to recover isolates from extreme environments like the ones harbored in the Altiplano highlands microbiome of Chile.

Taxonomic comparisons between culturable microbiota and metabarcoding data showed overrepresentation and underrepresentation of different taxa that could grow in culture, revealing several candidates that may provide support for the biotechnological ability of soil bacterial species living in a stressful environment that is highly challenging for life.

## Author Contributions

FM and JV-D wrote the manuscript. JM performed the bioinformatic analyses. FM performed the bacterial isolation. AG performed the PGP experiments. FM, JM, DM, MG, and VC designed and conceptualized the study.

### Conflict of Interest Statement

The authors declare that the research was conducted in the absence of any commercial or financial relationships that could be construed as a potential conflict of interest.

## Footnotes

^1^FigTree Software Available online at: http://tree.bio.ed.ac.uk/software/figtree/
