# Supplementary material for: Soil Bacterial Communities From the Chilean Andean Highlands: Taxonomic Composition and Culturability
Source: Front Bioeng Biotechnol. 2019 Feb 5;7:10. doi: 10.3389/fbioe.2019.00010 (PMC6371850; doi:10.3389/fbioe.2019.00010)
Supplement: Supplementary file 1 [file Data_Sheet_1.docx]

Supplementary Material

Soil bacterial communities from the Chilean Andean highlands: taxonomic composition and culturability

Felipe Maza^1^, Jonathan Maldonado^1,2^, Javiera Vásquez-Dean^1^, Dinka Mandakovic^1,2^, Alexis Gaete^1,2^, Verónica Cambiazo^1,2^, Mauricio González^1,2*^

***Correspondence:** Mauricio González mgonzale@inta.uchile.cl

**Supplementary Figures**

**Supplementary Figure 1:** Phylogenetic tree of bacterial sequences recovered by NGS from the highlands of the Atacama Desert.

**Supplementary Tables**

**Supplementary Table S1:** List of bacterial isolates, isolation site and culture medium used.

**Supplementary Table S2:** Biotechnological functions described in published articles for each isolate.

**Supplementary Table S3:** Microbial community diversity in different arid and hyper-arid deserts.

**Supplementary Table S4:** Average measured physicochemical characteristics of the desert soil of different studies compared to this study.

**Supplementary Table S5**: Average measured physicochemical characteristics of the desert soil of different studies compared to this study.


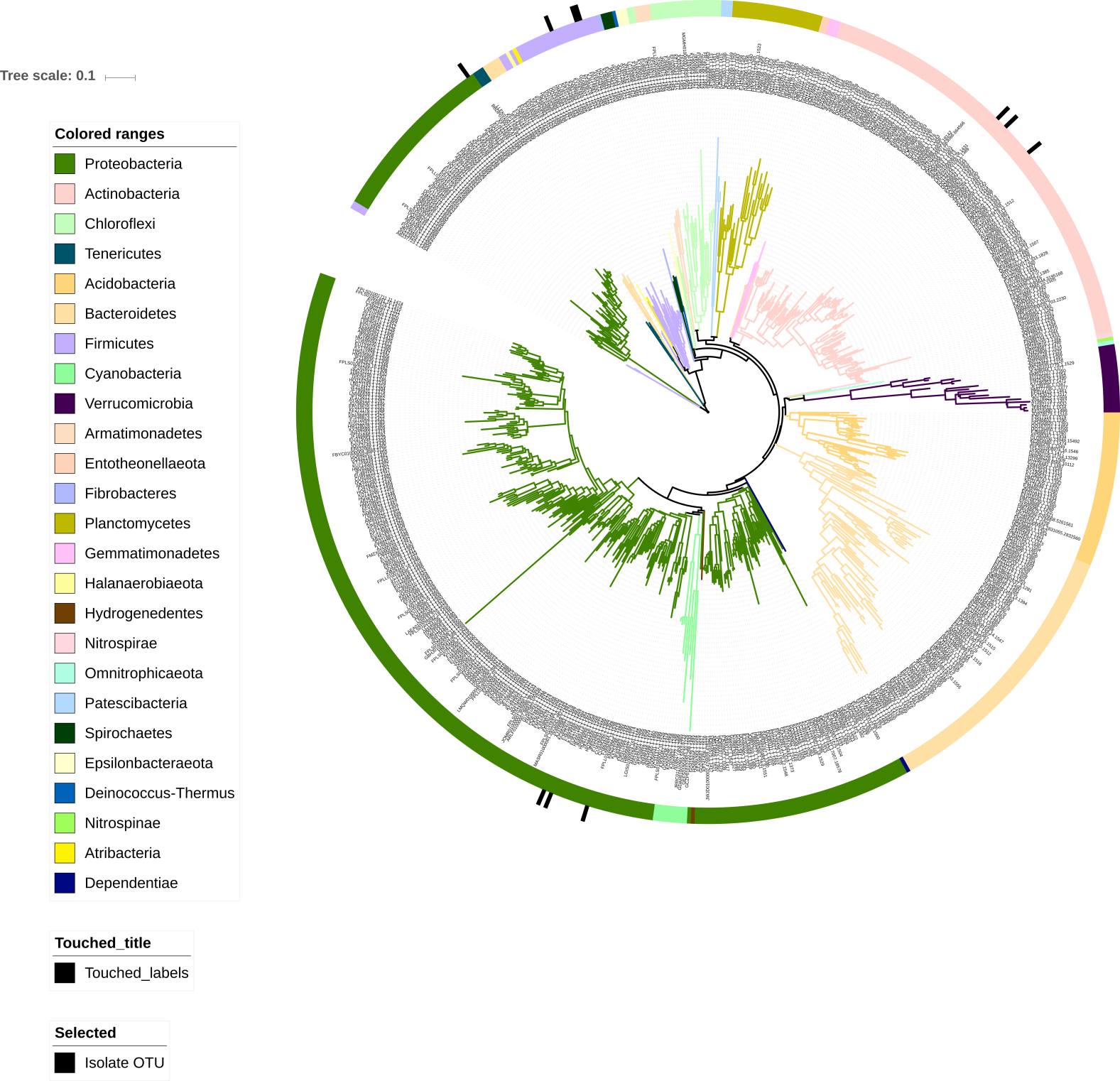

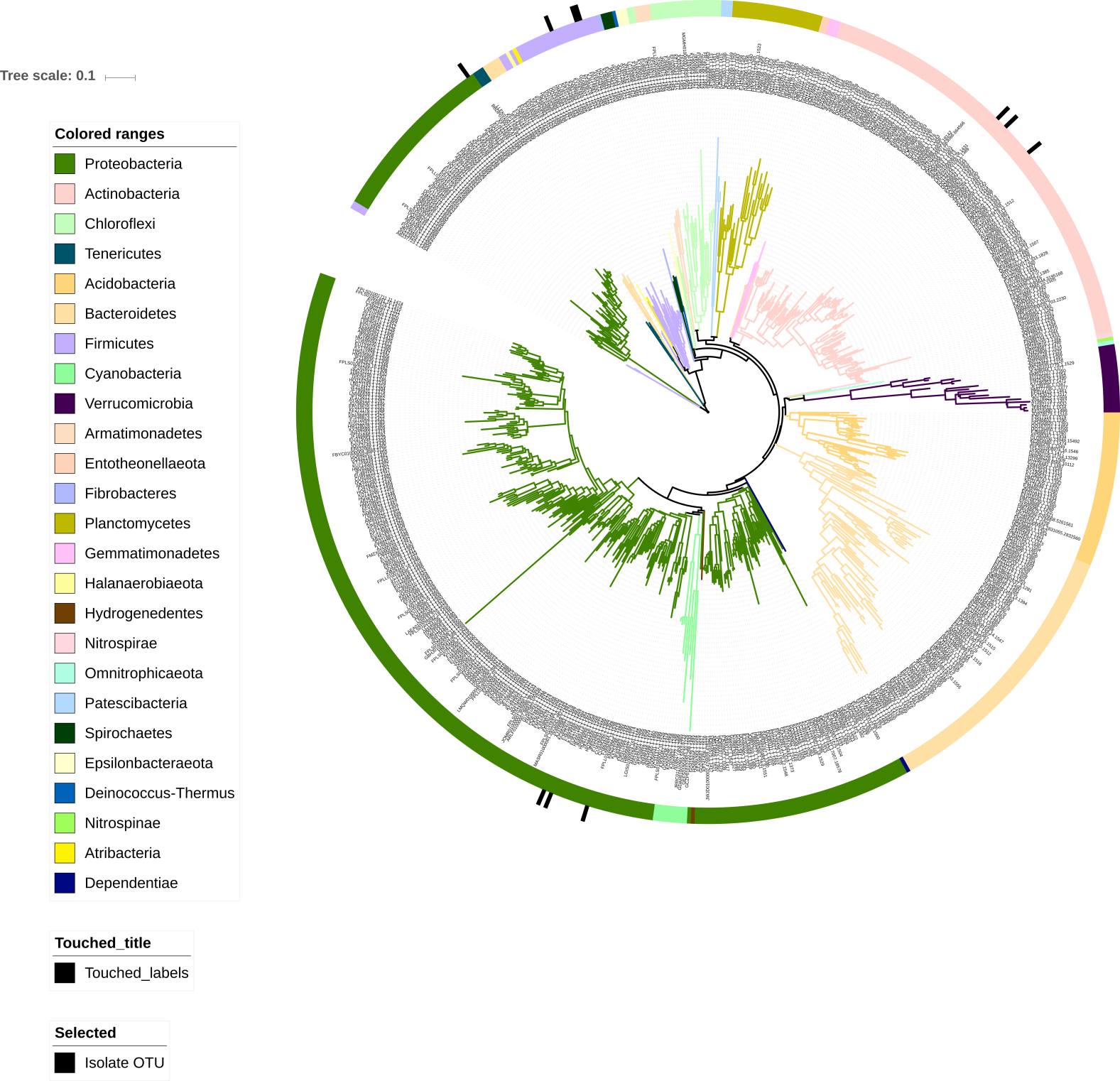

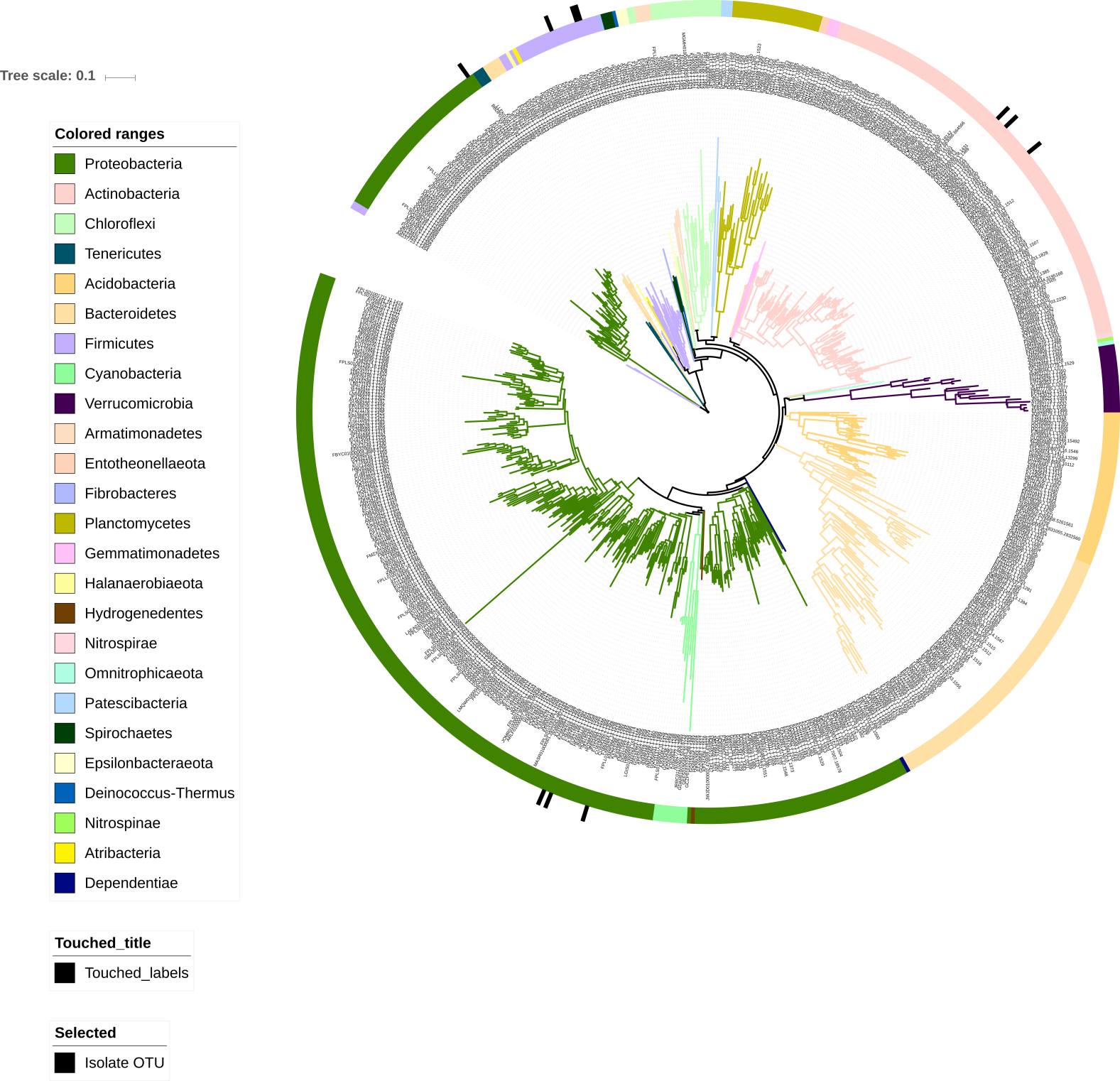


**Supplementary Figure 1**: Phylogenetic tree of bacterial sequences recovered by NGS from the highlands of the Atacama Desert. Phylogenetic tree produced by pruning the Silva 16S reference tree down to those tips representing OTUs found (n = 4,775) collapsed to genera (n = 480), after a quality filter of 0.005%. Colors indicate the assigned taxonomy (phyla) from the Silva database. Outermost circle indicates the location of isolated OTUs (n = 87) collapsed to genera (n = 10).

**Supplementary table 1**: List of bacterial isolates, isolation site and culture medium used.

| **Isolate** | **Isolation site** | **Isolation culture medium** | **Isolation culture condition** | **Closest species in RDP** |
| --- | --- | --- | --- | --- |
| *Microbacterium* sp. CGR2 | LLS | SEM | 25 °C, O_2_ | *Microbacterium paraoxydans* |
| *Bacillus* sp. ALS1 | LLS | SEM | 25 °C, O_2_ | *Bacillus simplex* |
| *Streptomyces* sp. ALS2 | LLS | SEM | 25 °C, O_2_ | *Streptomyces setonii* |
| *Streptomyces* sp. ALS3 | LLS | SEM | 25 °C, O_2_ | *Streptomyces anulatus* |
| *Carnobacterium* sp. ALS4 | LLS | SEM | 25 °C, O_2_ | *Carnobacterium pleistocenium* |
| *Paenibacillus* sp. ALS5 | LLS | SEM | 25 °C, O_2_ | *Paenibacillus xylanexedens* |
| *Streptomyces* sp. ALS6 | LLS | SEM | 25 °C, O_2_ | *Streptomyces* *setonii* |
| *Planococcus* sp. ALS7 | LLS | SEM | 25 °C, O_2_ | *Planococcus rifietoensis* |
| *Planococcus* sp. ALS8 | LLS | SEM | 25 °C, O_2_ | *Planococcus antarcticus* |
| *Halomonas* sp. ALS9 | LLS | SEM | 25 °C, O_2_ | *Halomonas alkaliantarctica* |
| *Halomonas* sp. ALS10 | LLS | SEM | 25 °C, O_2_ | *Halomonas alkaliantarctica* |
| *Arthrobacter* sp. AVS 13 | TLT1 | LB-SEM | 30 °C, O_2_ | *Arthrobacter globiformis* |
| *Arthrobacter* sp. AVS 27 | TLT1 | LB-SEM | 15 °C, O_2_ | *Arthrobacter oryzae* |
| *Arthrobacter* sp. AVS 28 | TLT1 | LB-SEM | 15 °C, O_2_ | *Arthrobacter scleromae* |
| *Arthrobacter* sp. AVS 29 | TLT1 | LB-SEM | 15 °C, O_2_ | *Arthrobacter aurescens* |
| *Arthrobacter* sp. AVS 32 | TLT1 | LB-SEM | 15 °C, O_2_ | *Arthrobacter aurescens* |
| *Arthrobacter* sp. AVS 33 | TLT1 | LB-SEM | 15 °C, O_2_ | *Arthrobacter pascens* |
| *Arthrobacter* sp. AVS 34 | TLT1 | LB-SEM | 15 °C, O_2_ | *Arthrobacter oryzae* |
| *Arthrobacter* sp. AVS 57 | TLT1 | LB-SEM | 15 °C, O_2_ | *Arthrobacter oryzae* |
| *Arthrobacter* sp. AVS 58 | TLT1 | LB-SEM | 15 °C, O_2_ | *Arthrobacter globiformis* |
| *Bacillus* sp. AVS 24 | TLT1 | LB-SEM | 30 °C, O_2_ | *Bacillus simplex* |
| *Bacillus* sp. AVS 19 | TLT1 | LB-SEM | 30 °C, O_2_ | *Bacillus simplex* |
| *Bacillus* sp. AVS 38 | TLT1 | 10% LB-SEM | 30 °C, O_2_ | *Bacillus simplex* |
| *Bacillus* sp. AVS 39 | TLT1 | 10% LB-SEM | 30 °C, O_2_ | *Bacillus simplex* |
| *Bacillus* sp. AVS 40 | TLT1 | 10% LB-SEM | 30 °C, O_2_ | *Bacillus simplex* |
| *Bacillus* sp. AVS 42 | TLT1 | 10% LB-SEM | 30 °C, O_2_ | *Bacillus simplex* |
| *Bacillus* sp. AVS 43 | TLT1 | 10% LB-SEM | 30 °C, O_2_ | *Bacillus simplex* |
| *Bacillus* sp. AVS 44 | TLT1 | 10% LB-SEM | 30 °C, O_2_ | *Bacillus simplex* |
| *Bacillus* sp. AVS 45 | TLT1 | 10% LB-SEM | 30 °C, O_2_ | *Bacillus simplex* |
| *Bacillus* sp. AVS 46 | TLT1 | 10% LB-SEM | 30 °C, O_2_ | *Bacillus simplex* |
| *Bacillus* sp. AVS 26 | TLT1 | LB-SEM | 15 °C, O_2_ | *Bacillus safensis* |
| *Bacillus* sp. AVS 49 | TLT1 | LB-SEM | 30 °C, O_2_ | *Bacillus simplex* |
| *Hymenobacter* sp. AVS 48 | TLT1 | 10% LB-SEM | 30 °C, O_2_ | *Hymenobacter aerophilus* |
| *Paenibacillus* sp. AVS 37 | TLT1 | LB-SEM | 15 °C, No-O_2_ | *Paenibacillus terrae* |
| *Paenibacillus* sp. AVS 35 | TLT1 | LB-SEM | 30 °C, No-O_2_ | *Paenibacillus pabuli* |
| *Paenibacillus* sp. AVS 36 | TLT1 | LB-SEM | 30 °C, No-O_2_ | *Paenibacillus pabuli* |
| *Pseudomonas* sp. AVS 23 | TLT1 | LB-SEM | 30 °C, O_2_ | *Pseudomonas brassicacearum* |
| *Pseudomonas* sp. AVS 25 | TLT1 | LB-SEM | 15 °C, O_2_ | *Pseudomonas frederiksbergensis* |
| *Pseudomonas* sp. AVS 31 | TLT1 | LB-SEM | 15 °C, O_2_ | *Pseudomonas frederiksbergensis* |
| *Streptomyces* sp. AVS 11 | TLT1 | LB-SEM | 30 °C, O_2_ | *Streptomyces vinaceus* |
| *Streptomyces* sp. AVS 21 | TLT1 | LB-SEM | 30 °C, O_2_ | *Streptomyces avidinii* |
| *Streptomyces* sp. AVS 22 | TLT1 | LB-SEM | 30 °C, O_2_ | *Streptomyces vinaceus* |
| *Streptomyces* sp. AVS 12 | TLT1 | LB-SEM | 30 °C, O_2_ | *Streptomyces vinaceus* |
| *Streptomyces* sp. AVS 14 | TLT1 | LB-SEM | 30 °C, O_2_ | *Streptomyces vinaceus* |
| *Streptomyces* sp. AVS 15 | TLT1 | LB-SEM | 30 °C, O_2_ | *Streptomyces ederensis* |
| *Streptomyces* sp. AVS 16 | TLT1 | LB-SEM | 30 °C, O_2_ | *Streptomyces subrutilus* |
| *Streptomyces* sp. AVS 17 | TLT1 | LB-SEM | 30 °C, O_2_ | *Streptomyces cirratus* |
| *Streptomyces* sp. AVS 18 | TLT1 | LB-SEM | 30 °C, O_2_ | *Streptomyces canus* |
| *Streptomyces* sp. AVS 50 | TLT1 | LB-SEM | 30 °C, O_2_ | *Streptomyces spororaveus* |
| *Streptomyces* sp. AVS 51 | TLT1 | LB-SEM | 30 °C, O_2_ | *Streptomyces spororaveus* |
| *Streptomyces* sp. AVS 52 | TLT1 | LB-SEM | 30 °C, O_2_ | *Streptomyces sp. QLS83* |
| *Streptomyces* sp. AVS 53 | TLT1 | LB-SEM | 30 °C, O_2_ | *Streptomyces spiramyceticus* |
| *Streptomyces* sp. AVS 61 | TLT1 | 10% LB-SEM | 30 °C, O_2_ | *Streptomyces chryseus* |
| *Streptomyces* sp. AVS 55 | TLT1 | LB-SEM | 15 °C, O_2_ | *Streptomyces vinaceus* |
| *Streptomyces* sp. AVS 56 | TLT1 | LB-SEM | 15 °C, O_2_ | *Streptomyces sp. QLS83* |
| *Arthrobacter* sp. ASTS 5 | TLT1 | LB-SEM | 30 °C, O_2_ | *Arthrobacter oryzae* |
| *Arthrobacter* sp. ASTS 11 | TLT1 | 10% LB-SEM | 30 °C, O_2_ | *Arthrobacter pascens* |
| *Arthrobacter* sp. ASTS 6 | TLT1 | LB-SEM | 15 °C, O_2_ | *Arthrobacter pascens* |
| *Arthrobacter* sp. ASTS 7 | TLT1 | LB-SEM | 15 °C, O_2_ | *Arthrobacter scleromae* |
| *Arthrobacter* sp. ASTS 8 | TLT1 | LB-SEM | 15 °C, O_2_ | *Arthrobacter pascens* |
| *Bacillus* sp. ASTS 1 | TLT1 | LB-SEM | 30 °C, O_2_ | *Bacillus simplex* |
| *Bacillus* sp. ASTS 2 | TLT1 | LB-SEM | 30 °C, O_2_ | *Bacillus simplex* |
| *Pseudomonas* sp. ASTS 4 | TLT1 | LB-SEM | 30 °C, O_2_ | *Pseudomonas brassicacearum* |
| *Variovorax* sp. ASTS 12 | TLT1 | 10% LB-SEM | 30 °C, O_2_ | *Variovorax soli* |
| *Streptomyces* sp. ASTS 10 | TLT1 | 10% LB-SEM | 30 °C, O_2_ | *Streptomyces nitrosporeus* |
| *Arthrobacter* sp. APUS 11 | TLT8 | LB-SEM | 15 °C, O_2_ | *Arthrobacter pascens* |
| *Bacillus* sp. APUS 1 | TLT8 | LB-SEM | 30 °C, O_2_ | *Bacillus megaterium* |
| *Bacillus* sp. APUS 2 | TLT8 | LB-SEM | 30 °C, O_2_ | *Bacillus megaterium* |
| *Bacillus* sp. APUS 3 | TLT8 | LB-SEM | 30 °C, O_2_ | *Bacillus drentensis* |
| *Bacillus* sp. APUS 32 | TLT8 | 10% LB-SEM | 30 °C, O_2_ | *Bacillus simplex* |
| *Bacillus* sp. APUS 33 | TLT8 | 10% LB-SEM | 30 °C, O_2_ | *Bacillus simplex* |
| *Bacillus* sp. APUS 36 | TLT8 | 10% LB-SEM | 30 °C, O_2_ | *Bacillus simplex* |
| *Bacillus* sp. APUS 37 | TLT8 | 10% LB-SEM | 30 °C, O_2_ | *Bacillus simplex* |
| *Bacillus* sp. APUS 41 | TLT8 | 10% LB-SEM | 30 °C, O_2_ | *Bacillus subtilis* |
| *Bacillus* sp. APUS 5 | TLT8 | LB-SEM | 15 °C, O_2_ | *Bacillus megaterium* |
| *Bacillus* sp. APUS 8 | TLT8 | LB-SEM | 15 °C, O_2_ | *Bacillus megaterium* |
| *Bacillus* sp. APUS 9 | TLT8 | LB-SEM | 15 °C, O_2_ | *Bacillus aryabhattai* |
| *Bacillus* sp. APUS 10 | TLT8 | LB-SEM | 15 °C, O_2_ | *Bacillus aryabhattai* |
| *Bacillus* sp. APUS 29 | TLT8 | LB-SEM | 15 °C, No-O_2_ | *Bacillus niacini* |
| *Bacillus* sp. APUS 22 | TLT8 | LB-SEM | 15 °C, No-O_2_ | *Bacillus bataviensis* |
| *Bacillus* sp. APUS 26 | TLT8 | LB-SEM | 15 °C, No-O_2_ | *Bacillus arbutinivorans* |
| *Bacillus* sp. APUS 19 | TLT8 | LB-SEM | 30 °C, No-O_2_ | *Bacillus bataviensis* |
| *Bacillus* sp. APUS 20 | TLT8 | LB-SEM | 30 °C, No-O_2_ | *Bacillus mycoides* |
| *Chryseobacterium* sp. APUS 40 | TLT8 | 10% LB-SEM | 30 °C, O_2_ | *Chryseobacterium soldanellicola* |
| *Paenibacillus* sp. APUS 6 | TLT8 | LB-SEM | 15 °C, O_2_ | *Paenibacillus pabuli* |
| *Paenibacillus* sp. APUS 24 | TLT8 | LB-SEM | 15 °C, No-O_2_ | *Paenibacillus odorifer* |
| *Paenibacillus* sp. APUS 15 | TLT8 | LB-SEM | 30 °C, No-O_2_ | *Paenibacillus pabuli* |
| *Paenibacillus* sp. APUS 42 | TLT8 | LB-SEM | 30 °C, No-O_2_ | *Paenibacillus lautus* |
| *Rhodococcus* sp. APUS 4 | TLT8 | LB-SEM | 30 °C, O_2_ | *Rhodococcus erythropolis* |
| *Rhodococcus* sp. APUS 35 | TLT8 | 10% LB-SEM | 30 °C, O_2_ | *Rhodococcus qingshengii* |
| *Rhodococcus* sp. APUS 13 | TLT8 | LB-SEM | 15 °C, O_2_ | *Rhodococcus erythropolis* |
| *Stenotrophomonas* sp. APUS 31 | TLT8 | 10% LB-SEM | 30 °C, O_2_ | *Stenotrophomonas maltophilia* |
| *Streptomyces* sp. APUS 7 | TLT8 | LB-SEM | 15 °C, O_2_ | *Streptomyces netropsis* |

LLS: Lejía Lake soil (Mandakovic et al., 2018a); TLT: Talabre-Lejía Transect; SEM: Soil Extract Medium; LB: Lysogeny Broth; O_2_: aerobic conditions; No-O_2_: anaerobic conditions.

**Supplementary table 2**: PGP activity of 12 isolates randomly selected.

| **Isolation Location** | **Strain** | **Isolate Identification** | **S. prod** | **IAA-prod** | **N-fix** | **ACC-dea** | **P-sol** |
| --- | --- | --- | --- | --- | --- | --- | --- |
| LLS | *Planococcus* sp. ALS8 | *Planococcus antarcticus* |  |  |  |  |  |
| LLS | *Planococcus* sp. ALS7 | *Planococcus rifietoensis* |  |  |  |  |  |
| LLS | *Streptomyces* sp. ALS6 | *Streptomyces setonii* |  |  |  |  |  |
| LLS | *Microbacterium* sp. CGR2 | *Microbacterium paraoxydans* |  |  |  |  |  |
| LLS | *Bacillus* sp. ALS1 | *Bacillus simplex* |  |  |  |  |  |
| L8 | *Bacillus*_APUS41 | *Bacillus subtilis* |  |  |  |  |  |
| L1 | *Pseudomonas*_ASTS4 | *Pseudomonas brassicacearum* |  |  |  |  |  |
| L8 | *Stenotrophomonas*_APUS31 | *Stenotrophomonas maltophilia* |  |  |  |  |  |
| L8 | *Paenibacillus*_APUS6 | *Paenibacillus pabuli* |  |  |  |  |  |
| L1 | *Arthrobacter*_AVS27 | *Arthrobacter oryzae* |  |  |  |  |  |
| L1 | *Bacillus*_AVS49 | *Bacillus simplex* |  |  |  |  |  |
| L8 | *Rhodococcus*_APUS4 | *Rhodococcus erythropolis* |  |  |  |  |  |

* S. prod: siderophore production; IAA-prod: indole acetic acid production; N-fix: nitrogen fixation; ACC-dea: ACC (1-Aminocyclopropane-1-Carboxylate) deaminase activity; P-sol: phosphate solubilization. Black squares indicate positive activity to the mentioned test.

**Supplementary table 3**: Biotechnological functions described in published articles for each isolate.

| Putative bacterial species | Biotechnological category | NCBI accession number |
| --- | --- | --- |
| *Arthrobacter globiformis* | PGP | 26632776 |
| *Arthrobacter pascens* | PGP | 29389906 |
| *Bacillus arbutinivorans* | PGP | 21077114 |
| *Bacillus aryabhattai* | Bioremediation | 27619063 |
| *Bacillus aryabhattai* | PGP | 28377746 |
| *Bacillus aryabhattai* | PGP | 28248011 |
| *Bacillus drentensis* | Bioremediation | 18522454 |
| *Bacillus drentensis* | Bioremediation | 25199604 |
| *Bacillus drentensis* | PGP | 27379151 |
| *Bacillus megaterium* | Bioremediation | 19447212 |
| *Bacillus megaterium* | PGP | 24101562 |
| *Bacillus mycoides* | Antibiotic Production | 29124466 |
| *Bacillus mycoides* | Antibiotic Production | 26465807 |
| *Bacillus mycoides* | Antibiotic Production | 19898544 |
| *Bacillus mycoides* | Bioremediation | 15712752 |
| *Bacillus mycoides* | PGP | 30051589 |
| *Bacillus niacini* | PGP | 21077114 |
| *Bacillus safensis* | Antibiotic Production | 29167801 |
| *Bacillus safensis* | Antibiotic Production | 29463889 |
| *Bacillus safensis* | Bioremediation | 28179901 |
| *Bacillus safensis* | PGP | 28868579 |
| *Bacillus safensis* | PGP | 23239372 |
| *Bacillus simplex* | Antibiotic Production | 19259714 |
| *Bacillus simplex* | Bioremediation | 18522454 |
| *Bacillus simplex* | PGP | 21157636 |
| *Bacillus simplex* | PGP | 26442090 |
| *Bacillus simplex* | PGP | 29751738 |
| *Halomonas* | Bioremediation | 24795705 |
| *Halomonas* | Bioremediation | 20172021 |
| *Microbacterium* | Antibiotic Production | 29567053 |
| *Microbacterium* | Bioremediation | 28956196 |
| *Microbacterium* | PGP | 29253319 |
| *Paenibacillus lautus* | Bioremediation | 15712752 |
| *Paenibacillus pabuli* | PGP | 29404231 |
| *Paenibacillus pabuli* | PGP | 29906776 |
| *Paenibacillus terrae* | Antibiotic Production | 25908148 |
| *Paenibacillus terrae* | Antibiotic Production | 24382692 |
| *Pseudomonas brassicacearum* | PGP | 29905506 |
| *Pseudomonas frederiksbergensis* | Bioremediation | 29384200 |
| *Pseudomonas frederiksbergensis* | Bioremediation | 29169219 |
| *Pseudomonas frederiksbergensis* | Bioremediation | 25522518 |
| *Rhodococcus erythropolis* | Antibiotic Production | 29980745 |
| *Rhodococcus erythropolis* | Bioremediation | 29879458 |
| *Rhodococcus erythropolis* | PGP | 28232452 |
| *Rhodococcus qingshengii* | Antibiotic Production | 18048720 |
| *Rhodococcus qingshengii* | Bioremediation | 25401077 |
| *Stenotrophomonas maltophilia* | Antibiotic Production | 27446008 |
| *Stenotrophomonas maltophilia* | Antibiotic Production | 27446008 |
| *Stenotrophomonas maltophilia* | Bioremediation | 19940938 |
| *Stenotrophomonas maltophilia* | PGP | 27446008 |
| *Streptomyces avidinii* | Antibiotic Production | 6933515 |
| *Streptomyces canus* | Antibiotic Production | 5004673 |
| *Streptomyces canus* | Antibiotic Production | 23360202 |
| *Streptomyces canus* | Antibiotic Production | 24568288 |
| *Streptomyces canus* | Antibiotic Production | 26043159 |
| *Streptomyces cirratus* | Antibiotic Production | 6833148 |
| *Streptomyces ederensis* | Antibiotic Production | 18488260 |
| *Streptomyces ederensis* | Antibiotic Production | 29705162 |
| *Streptomyces netropsis* | Antibiotic Production | 25415678 |
| *Streptomyces nitrosporeus* | Antibiotic Production | 14897502 |
| *Streptomyces nitrosporeus* | Antibiotic Production | 7319904 |
| *Streptomyces spiramyceticus* | Antibiotic Production | 1773014 |
| *Streptomyces subrutilus* | Bioremediation | 11150672 |
| *Streptomyces vinaceus* | Antibiotic Production | 6163840 |
| *Streptomyces vinaceus* | Antibiotic Production | 19053507 |
| *Streptomyces vinaceus* | PGP | 19937030 |
| *Variovorax soli* | Bioremediation | 28011373 |
| *Variovorax soli* | Bioremediation | 25662244 |

**Supplementary table 4**: Microbial community diversity in different arid and hyper-arid deserts.

| Phylum | Our study | Fierer 2012 (Hot) | Fierer 2012 (Cold) | McCann 2016 | Pointing 2009 | Costello 2009 |
| --- | --- | --- | --- | --- | --- | --- |
| Proteobacteria | 40.5% | 19.7% | 11% | 32% | 4% | 4.3% |
| Acidobacteria | 19.6% | 13% | 15.7% | 5.8% | 31% | 22.4% |
| Actinobacteria | 16.7% | 16.6% | 24.7% | 21.6% | 33% | 32.6% |
| Bacteroidete | 5.8% | 13.7% | 19.3% | 5.4% | 2% | 14.9% |
| Firmicutes | 3% | 0.6% | 0.1% | 1.4% | ND | ND |
| Chloroflexi | 2% | 13.7% | 19.3% | 12.8% | 3% | 5.1% |

*ND: No data.

**Supplementary table 5**: Average measured physicochemical characteristics of the desert soil of different studies compared to this study.

|  | Our study | Fierer 2012 (Hot) | Fierer 2012 (Cold) | McCann 2016 | Pointing 2009 | Costello 2009 |
| --- | --- | --- | --- | --- | --- | --- |
| Location | Altiplano highlands (Atacama Desert) | Southwestern deserts USA | Antarctic dry valleys | Kongsfjorden (Polar desert) | Antarctic dry valleys | Volcán Socompa (Atacama Desert) |
| Aridity Index | 0.2 | 0.2 | 0.05 | 0.2 | 0.2 | 0.2 |
| pH | 6.2 | 8.1 | 9 | 6.8 | 8.7 | 5.23 |
| MAT (°C) | 5.6 | 15.8 | -19 | -4.6 ^†^ | -16.2^†^ | -5 |
| MAP (mm) | 109 | 208 | 100 | 191 | 184^‡^ | <200 |
| Altitude (m a.s.l.) | 4,135 | 1,315 | 147 | 15 | 430* | 5,235 |

^†^(Førland et al., 2011). ^‡^www.scar.org. *www.geoplaner.com
